# Mental health, learning behaviour and perceived fatigue among university students during the COVID-19 outbreak: a cross-sectional multicentric study in the UAE

**DOI:** 10.1186/s40359-022-00758-z

**Published:** 2022-03-02

**Authors:** Sultan M. Mosleh, Raed M. Shudifat, Heyam F. Dalky, Mona M. Almalik, Malek K. Alnajar

**Affiliations:** 1 Faculty of Health Science, Higher Colleges of Technology, PO Box 1626, Fujairah, UAE; 2 Faculty of Health Science, Higher Colleges of Technology, Sharjah, UAE; 3 Faculty of Nursing, Mutah University, Karak, Jordan; 4 Faculty of Nursing, Jordan University of Science and Technology, Irbid, Jordan

**Keywords:** COVID-19 outbreak, Psychological well-being, Fatigue level, Home confinement-related stress

## Abstract

**Background:**

The rapid shift to online education due to COVID-19 quarantine challenged students’ ability to accept pure online learning without negative consequences for their physical, emotional and mental health. Some educational institutions introduced new strategies to reduce the psychosocial burden associated with online learning during home confinement. Thus, the primary aims were to determine the consequences of COVID-19 for the psychological well-being and fatigue levels of higher education students and to explore the effects of a new academic assessment approach in reducing home confinement stress.

**Method:**

A cross-sectional online survey was conducted among students, from 30 August to 30 September 2020, of 7 disciplines in all 16 higher colleges of technology in the United Arab Emirates (UAE). The Mental Well-being and Learning Behaviours Scale and the modified Copenhagen Burnout Inventory were used to evaluate students’ psychological well-being and fatigue levels. A Welch t-test and Welch ANOVA were performed to determine the differences in perceived psychological well-being associated with students’ characteristics. Second, Kruskal_Wallis and Mann_Whitney were performed to determine the differences in fatigue level based on students’ characteristics.

**Results:**

One thousand four hundred students participated. The majority were female (78.5%) and aged from 21–25 years (58.1%). Around 14% of respondents were married with children. Nearly 40% were satisfied with the new assessment approach introduced during the COVID pandemic and 45.5% perceived it as having reduced their home confinement stress. The mean psychological distress score of 3.00 (SD ± 0.71) indicates a moderate impact of COVID-19 on psychological well-being. Students’ psychological distress was positively correlated with fatigue level (0.256, *p* < 0.001) and negatively correlated with the perceived impact of the new assessment approach on student lifestyle (− 0.133, *p* < 0.001), physical health (− 0.149, *p* < 0.001) and coping with stress (− 0.125, *p* < 0.001). Male students experienced significantly lower fatigue and better psychological well-being than female students.

**Conclusion:**

The study reveals that new assessment approaches which emerged during home confinement reduced students’ perception of stress and of impaired lifestyle. However, students still had a considerable burden of psychological distress, requiring further preventive measures to maintain their psychological well-being during future outbreak events. Educational institutions should consider additional strategies to improve students’ preparedness for online teaching, which could help maintain their psychological well-being.

## Background

Since the emergence of Coronavrus Disease 2019 (COVID-19) in Wuhan in December 2019, major extraordinary measures have been implemented globally to reduce transmission of the Severe Acute Respiratory Syndrome Coronavirus 2 (SARS-CoV-2 virus) [1, 2]. Despite these, within weeks the ensuing pandemic caused significant changes to the educational landscape worldwide. In the United Arab of Emirates (UAE), the first case of COVID-19 was identified on 29 January 2020. In response, the government has taken stringent steps to control the spread of the virus, including stay-home orders of two to three months duration [1, 3]**.** On 3 March 2020, the UAE Ministry of Education announced that all schools, colleges and universities across the country would close for a month starting Sunday 8 March as part of efforts to contain the spread of the virus [4].

In response to the spread of COVID-19, higher education institutions (HEIs) around the globe closed their physical campuses and moved rapidly from traditional face-to-face courses to remote or online courses [5, 6]. According to the United Nations Educational, Scientific and Cultural Organization [7], the pandemic has interrupted the learning of more than one billion students in 129 countries worldwide. In the UAE, many HEIs have been forced to make timely adjustments to ensure the provision of education and sustain students’ academic progress.

Consequently, students are liable to have experienced stressors subsequent to the academic burden, lack of relaxation time and repeated examinations in a competitive environment [8, 9]. The pandemic disrupted student life; normal classes were suspended and students could not participate in social activities, affecting their academic performance and aggravating stress levels [1, 10, 11]. Previous studies have found significantly high stress and anxiety levels among university and college students during the pandemic, negatively impacting their perception of online courses [10, 12–14].

Previous research has established that students’ ability to use learning technology tools, lack of IT knowledge, lack of technical support during virtual classes and the inappropriateness of online course content were causes of students’ distress and burden during home quarantine [10]. Hasan and Bao [1] examined the effects on students’ psychological distress of ‘e-learning crack-up’ during COVID-19 lockdown; their survey of 400 students identified perception of e-learning crack-up as a significant independent predictor of psychological distress.

The prevalence of emotional distress varies among recent studies but demonstrates that a considerable percentage of students risk psychological distress. For example, Li et al. [15] found that 139 (13.63%) had anxiety related to the COVID-19 pandemic and 406 (39.80%) of them had high stress levels. In a Spanish study, 34.19% of respondents showed moderate to extremely severe depression symptoms, 21.34% showed extremely severe anxiety symptoms and 28.14% exhibited moderate to severe stress symptoms subsequent to the COVID-19 pandemic [16]. The literature also identifies various factors influencing students’ mental health. A recent study among 7143 college students revealed that COVID-19-related factors such economic stressors, effects on daily life and academic delays were positively associated with the level of anxiety symptoms [10], while supportive family, living with parents and having a steady income were protective against high levels of anxiety. Moreover, the findings of Meo et al. [17] show that a sense of emotional detachment from family, friends and fellow students was significantly associated with emotional distress and had negatively impacted students’ academic performance and total daily studying hours. Despite the extensive research conducted over the last year, however, no attention appears to have been paid to changes in assessment methods during the crisis, students’ GPA before and after the outbreak and marital status. This study therefore explores the association of these factors with students’ fatigue levels and psychological well-being.

### Significance of the study

Before 2020, a major transition to online education was already underway. Over the past decades, online teaching had become a strategic objective for many HEIs, including those in the UAE. Then, when the pandemic nature of the COVID-19 outbreak became evident in the spring of 2020, universities abruptly went online with very few days of planning [18]. A growing body of literature recognises the importance of exploring the impact of the pandemic and the sudden shift to online learning on students’ psychological well-being. To our knowledge, no studies within Arab culture and the Gulf countries have examined this impact. The study also aims to determine the association of students’ characteristics with perceived psychological well-being and the fatigue associated with confinement due to the COVID-19 outbreak.

The Higher Colleges of Technology (HCT) is a federal educational institution comprising the largest student body in the UAE, with more than 20,000 students in 16 campuses across the country. Since March 2020, various strategies have been actioned to ensure a smooth transmission to online learning and teaching experience [3]. One of strategies was to modify the academic assessment approach after extensive consultation with faculty and the endorsement of the HCT’s Academic Council. Examples of changes to traditional exams include splitting them into multiple online quizzes and assignments; replacing them with projects or case studies, reducing the weight to 15% and distributing the remaining weight over other formative assessments; replacing them with e-portfolios; using questions with an increased level of cognitive complexity; and setting online, open-book practical exams (timed and scheduled during online class sessions).

This study examines three main hypotheses: (1) The abrupt transition to pure online learning increased students’ psychological distress and fatigue; (2) The modified assessment strategy during home confinement moderated the effect on students’ family lifestyles, physical health, mental health and stress; and (3) Students’ perception of psychological well-being moderated fatigue levels and academic performance.

The results could assist colleges, administrators and policymakers in the UAE by providing a theoretical basis for assessing psychological well-being and identifying the appropriate actions to help students during future pandemics. It should also provide guidelines for policymakers on possible mechanisms to moderate the impacts of anxiety on students during such health crises.

## Methods

### Design and setting

The study had a descriptive cross-sectional quantitative design. Data were collected at one point in time via an online survey sent to potential participants by email, to be completed at their convenience. The email was sent from 30 August 2020 to 30 September 2020, and up to three reminders were sent weekly to enhance the response rate. The target population was all 20,000 students on 16 HCT campuses across the UAE.

### Data collection

Data were collected over one month. The survey, which took the participants about 10–14 min to complete, contained questions on demographics, academic performance, psychological well-being and fatigue. The first section elicited information about students’ age, gender, marital status, academic level and Grade Point Average (GPA) before and after the pandemic semester. Participants also reported their perceptions of the impact of learning from home on their daily family lifestyle, physical health, mental health and ability to cope with stress. These four items were assessed on a scale from 1 (very negatively) to 5 (very positively). A single-item measure was used to reduce the demand on participants and make it easier to complete the entire survey.

Psychological well-being was evaluated using the Mental Well-being and Learning Behaviors scale [17], a five-point Likert scale from 1 (strongly disagree) to 5 (strongly agree). This instrument had two parts, the first comprising twelve questions to assess psychological well-being and stressors resulting from COVID-19, with a higher score indicating a greater negative impact on students’ well-being. The second part comprised eight items on learning behaviours during the COVID outbreak, with a higher score representing a more positive attitude to e-learning. In this study, the Cronbach’s alpha values for psychological well-being and learning behaviours were α = 0.90 (95%CI: 0.888 to 0.904) and α = 0.84 (95%CI: 0.833 to 0.856) respectively.

Student fatigue level related to learning online from home subsequent to the COVID-19 pandemic was evaluated by a modified version of the Copenhagen Burnout Inventory [19]. The modified scale comprised 16 items in three subscales, measuring personal burnout attributed by the participant to factors unrelated to work (5 items), to working online (5 items) and to learning from home (6 items). Total score range from 13 to 65, all items were scored on a four‐point Likert scale. The internal consistency of the three subscales was α = 0.93; (95%CI: 0.924 to 0.936), α = 0.81 (95%CI: 0.0.799 to 0.829) and α = 0.94; (95%CI: 0.941 to 0.950) respectively.

The instrument was piloted among 25 students to determine whether the items were clear and concise, before being administered to the full sample.

### Data analysis

The data were analysed quantitatively. All collected data were organized and summarized by descriptive statistics (measures of central tendency, dispersion and percentages) and the chi-square test was applied to determine how some demographic variables varied with the students’ knowledge and health outcomes. Mean and standard deviation were reported to represent the average score for each scale item. In all statistical tests, two-tailed tests of significance and confidence intervals were based on the unadjusted *P-value less than* 0.05 level. First, Psychological well-being mean score was normally distributed (skewness = − 0.050; kurtosis = 0.133). Equal variance was not assumed in all performed test due to the variation in sample size between the comparisons groups, therefore the Welch t-test and Welch ANOVA were performed to determine the differences in perceived psychological well-being based on students’ characteristics. The fatigue mean score was slightly skewed, Kruskal_Wallis and Mann_Whitney tests were performed to determine the differences in fatigue level based on students’ characteristics. The software used for data analysis was SPSS version 23 [19].

### Ethical considerations

This study was conducted with the approval of the Research and Ethical Committee of the Higher Colleges of Technology. Students signed an informed consent and informed that their participation was voluntary, and that they could withdraw from the study at any time. Data collection process was carried out in accordance with relevant guidelines and regulations.

## Results

One thousand four hundred students participated in the study. Most were female (78.5%) and aged 21–25 years (58.1%), while around 14% were married with children (Table 1).

### Impact of the COVID outbreak on academic performance

During COVID-19 quarantine, major coursework assessments were split into short quizzes and open-book exams. Almost 40% of participants reported being satisfied with the changes in assessment delivery method during home confinement and 45.5% perceived it as having reduced their stress. Three quarters of participants (76%) reported an improvement in their GPA during the pandemic semester and a similar percentage believed that online assessment had succeeded in reducing their stress levels. Projects and open-book exams (instead of the final written exam) were the assessment methods perceived as having contributed most to GPA improvement and stress reduction during COVID-19 quarantine (Table 2).

**Table 1:** Participants' demographics (N = 1431)

Variable		n (%)
Faculty	Business	366 (25.6)
	Computer information science	301 (21.0)
	Education	57 (4.0)
	Engineering	347 (24.2)
	Health sciences	278 (19.4)
	Military and security	6 (0.4)
	General academic require-ments division	76 (5.3)
Age in years	Under 20	532 (37.2)
	21–25	831 (58.1)
	26–30	49 (3.4)
	More than 30	19 (1.3)
Gender	Male	308 (21.5)
	Female	1123 (78.5)
Marital status	Married	120 (8.4)
	Single	1300 (90.8)
	Divorced	8 (.6)
	Widowed	3 (.2)
Number of children	None	1352 (94.5)
	1	45 (3.1)
	2	16 (1.1)
	3	9 (.6)
	4	3 (.2)
Work status	Employed full time	82 (5.7)
	Employed part time	40 (2.8)
	Unemployed	1309 (91.5)
Student level	Semester two	316 (22.1)
	Semester three	250 (17.5)
	Semester four	159 (11.1)
	Semester five	147 (10.3)
	Semester six	175 (12.2)
	Semester seven	182 (12.7)
	Semester eight	202 (14.1)

### Impact of new assessment approach on student stress level

Students reported diverse perceptions of the impact of modified assessment during the COVID-19 pandemic. A fifth (22%) of students reported a negative effect on family lifestyle, while more than half (53%) said this impact was positive (Table 3). Over a quarter (27.8%) reported a negative effect on physical health, while almost as many (27.1%) perceived negative mental health effects. As to stress, 28.8% of students felt that online assessment had increased their stress levels, whereas 45.5% felt that the modified assessment had reduced their stress.

**Table 2:** Impact of online teaching and modified assessment method on academic performance

Variables		n (%)
Has your GPA improved at the end of Spring 2020 semester?	Yes	1090 (76.2)
	No	341 (23.8)
Did the modified assessment reduce your stress associated with home confinement?	Yes	1082 (75.6)
	No	349 (24.4)
The most successful type of online assessment	Final exam	628 (43.9)
	Case study	470 (32.8)
	Project	715 (50)
	Presentation	647 (45.2)
	Test/quiz	693 (48.4)
	Written assignment	487 (34)
	Open book exam	650 (45.4)
Was the final exam replaced?	Yes	369 (25.8)
	No	1062 (74.2)
Which of the following were included in your course to replace an online Spring 2020 final exam?	Exam	139 (9.7)
	Case Study	242 (16.9)
	Project	184 (12.9)
	Presentation	126 (8.8)
	Test/quiz	108 (7.5)
	Written assignment	145 (10.1)
	Open book exam (non-FWA)	13 (.9)
The improvement in your GPA was due to:	Case Study	428 (29.9)
	Project	732 (51. 3)
	Presentation	552 (38.6)
	Test/quiz	555 (38.8)
	Written assignment	420 (29.6)
	Open book exam (non-FWA)	574 (40.1)

### Student fatigue during e-learning

The median fatigue score was with a interquartile range of 22 to 46, indicating moderate fatigue. On the personal, online learning and learning from home burnout subscales respectively, the median score were 2.20 (IQR 1.20 to 3.0), 2.20 (IQR 1.40 to 3.0), and 2.0 [1 to 3]. Results on all subscales indicate low fatigue levels associated with e-learning. However, a significant percentage of students reported always having fatigue more than five days per week.

### Students’ psychological well-being during the COVID pandemic and quarantine

Participants’ mean score on psychological well-being was 3.0/5 (SD = 0.71), with a range from 1 to 5, indicating a moderate impact. The mean score on the mental health subscale was 3.00 (SD = 0.87), with a range from 1 to 5, and approximately a third of students agreed that they were always thinking about being infected with the virus, with a mean of 3.07. Thirty-seven percent of students perceived themselves as depressed, while 31.1% agreed that they felt detached from social life, were anxious and suffered from insomnia, with a mean of 2.99.

On the learning behaviour subscale, the mean score was 3.01 (SD = 0.68), indicating a moderate impact of COVID-19 on learning behaviour. Lack of motivation was reported by 22.6% of students, while 18% said they suffered from poor concentration and 23.3% had difficulty remembering recent information. Almost 40% reported an increase in time spent studying, while 25% stated that this time had decreased.

Univariate coefficients for the association between the main study outcomes are presented in Table 4. A positive relationship emerges between fatigue and psychological distress, indicating that students who perceived a higher level of psychological distress also perceived higher fatigue levels. In addition, a higher impact on psychological well-being (mental health and learning behaviour) was observed among students who reported a low negative impact of the new assessment approach on their lifestyle, physical health and coping with stress.

**Table 3:** Impact of new assessment models on students (N = 1431)

Domain	Very negatively	Negatively	No effect	Positively	Very positively	M(SD)
Family lifestyle	113 (7.9)	211 (14.7)	353 (24.7)	328 (22.9)	426 (29.8)	3.52 (1.2)
Physical health	144 (10.1)	254 (17.7)	358 [25]	287 (20.1)	388 (27.1)	3.36 (1.3)
Mental health	146 (10.2)	242 (16.9)	345 (24.1)	307 (21.5)	391 (27. 3)	3.39 (1.3)
Stress levels	180 (12.6)	232 (16.2)	369 (25.8)	292 (20.4)	358 [25]	3.29 (1.3)

### Differences based on students’ demographical characteristics

Various sociodemographic variables significantly associated with study outcomes (Table 5). Male students experienced more significant psychological well-being than females. The student who was satisfied with the number of online assessments and had improved GPA at the end of the spring semester had more significant psychological well-being. The Welch ANOVA test revealed more significant psychological well-being among students with a GPA above two and are very satisfied with the number of online assessments. Health science students experienced better psychological well-being, but the result is less statistically significant.

**Table 4:** Correlation coefficient between psychological well-being, fatigue and stress

	Fatigue ^a^	Psychological distress	Satisfied with new assessments	Impact of new assessment on family lifestyle	Impact of new assessment on physical health
Psychological distress	.254**				
Satisfaction with new assessment approach	-.435**	-.060*			
Impact of new assessment on Family lifestyle	-.552**	-.133*	.459*		
Impact of new assessment on Physical health	-.564**	-.149*	.443*	.766*	
Impact of new assessment on ability to cope with stress	-.574**	-.125*	.451*	.723*	.723*

**Spearman Correlation *p* < 0.01, Pearson Correlation * p < 0.01

Results shown in Table 6 suggest significant differences in fatigue level based on student’s characteristics. Kruskal_Wallis test result showed that students were more significantly had less fatigue level if they were between 26 and 30 years old, single, employed in a full-time job, and were very satisfied with the number of assessments in the spring semester.

**Table 5:** Differences psychological well-being means scores based on students’ demographics (N = 1431)

Variable	Psychological well-being M (SD)	*P*-value
Gender		0.054
Male	2.94 (.75)	
Female	3.02 (.7)	
Faculty		0.277
Business	3.01 (.77)	
Computer information science & applied media	3 (.74)	
Education	3.11 (.68)	
Engineering technology and science	3 (.66)	
Health sciences	3.01 (.65)	
Military and security	2.28 (.89)	
General academic requirements division	2.97 (.71)	
Age in years		0.41
Less than 20	3 (.74)	
21–25	3 (.69)	
26–30	2.8 (.79)	
More than 30	3.22 (.53)	
Marital status		0.024
Married	2.9 (.7)	
Single	3 (.71)	
Divorced	2.73 (.42)	
Widowed	2.4 (.43)	
Number of children		0.63
None	3 (.71)	
1	2.8 (.69)	
2	2.9 (.61)	
3	2.96 (.95)	
4	3 (.39)	
Working status		0.67
Employed full time	3.07 (.89)	
Employed part time	2.98 (.67)	
Unemployed	3 (.7)	
Student level		0.54
Semester two	3 (.8)	
Semester three	2.97 (.71)	
Semester four	3.1 (.67)	
Semester five	3 (.75)	
Semester six	2.99 (.69)	
Semester seven	2.98 (.65)	
Semester eight	2.96 (.63)	
Current GPA		0.006
Not calculated (semester 2 students)	3.34 (.78)	
Below 1	3.34 (1.1)	
1 to 1.9	3.1 (.8)	
2 to 2.9	2.98 (.7)	
Above 3	2.98 (.69)	
Previous GPA		0.37
Not calculated (semester 2 students)	3.05 (.82)	
Below 1	3.41 (.43)	
1 to 1.9	2.97 (.74)	
2 to 2.9	2.98 (.71)	
Above 3	3.03 (.68)	
Satisfaction with the number of assessments		0.028
Very dissatisfied	2.99 (.84)	
Dissatisfied	3.06 (.68)	
Neutral	3.05 (.52)	
Satisfied	3.01 (.66)	
Very satisfied	2.89 (.92)	
Has your GPA improved at the end of Spring 2020 semester?		< .001
Yes	2.96 (.7)	
No	3.14 (.73)	
Was the type of online assessment successful?		< .001
Yes	2.97 (.7)	
No	3.12 (.72)	

*p-value is significant at ≤ 0.05 using Welch ANOVA test

**p-value is significant at ≤ 0.05 using Welch t-test

**Table 6:** Differences of fatigue means scores based on students’ demographics (N = 1431)

Variable	Median (interquartile range)	*P *value
Gender		0.001**
Male	1.65 (1.25–2.62)	
Female	2.31 (1.50–2.93)	
Faculty		0.078*
Business	1.93 (1.31–2.81)	
Computer information science & applied media	2.18 (1.40–2.93)	
Education	2.37 (1.65–2.93)	
Engineering technology and science	2.00 (1.37–2.81)	
Health sciences	2.50 (2.50–2.93)	
Military and security	2.21 (1.54–2.87)	
General academic requirements division	2.31 (1.39–3.00)	
Age in years		0.001*
Less than 20	2.31 (1.56–2.93)	
21–25	2.12 (1.37–2.87)	
26–30	1.56 (1.18–2.25)	
More than 30	1.81 (1.25–2.68)	
Marital status		0.019*
Married	1.90 (1.25–2.54)	
Single	2.25 (1.43–2.87)	
Divorced	2.00 (1.42–3.04)	
Widowed	1.18 (1.187–1.187)	
Number of children		0.165*
None	2.25 (1.43–2.87)	
1	1.81 (1.25–2.56)	
2	2.12 (1.23–2.60)	
3	1.18 (1.18–1.56)	
4	1.62 (1.125–1.62)	
Working status		0.031*
Employed full time	1.78 (1.29–2.68)	
Employed part time	2.40 (1.57–2.87)	
Unemployed	2.18 (1.40–2.87)	
Student level		0.078*
Semester two	2.37 (1.50–2.87)	
Semester three	2.31 (1.50–2.89)	
Semester four	2.18 (1.37–2.93)	
Semester five	2.18 (1.37–2.93)	
Semester six	2.00 (1.31–2.93)	
Semester seven	1.93 (1.35–2.93)	
Semester eight	2.12 (1.31–2.75)	
Current GPA		0.83*
Not calculated (semester 2 students)	2.59 (1.96–2.85)	
Below 1	2.43 (1.68–2.71)	
1 to 1.9	2.31 (1.48–2.90)	
2 to 2.9	2.00 (1.31–2.81)	
Above 3	2.25 (1.50–3.00)	
Previous GPA		0.001*
Not calculated (semester 2 students)	2.37 (1.50–2.87)	
Below 1	2.25 (1.56–2.59)	
1 to 1.9	1.96 (1.31–2.75)	
2 to 2.9	2.06 (1.31–2.76)	
Above 3	2.37 (1.56–3.06)	
Satisfaction with the number of assessments		0.001*
Very dissatisfied	2.87 (1.87–3.62)	
Dissatisfied	2.81 (2.25–3.32)	
Neutral	2.31 (1.68–2.87)	
Satisfied	1.87 (1.31–2.56)	
Very satisfied	1.37 (1.18–2.06)	
Has your GPA improved at the end of Spring 2020 semester?		0.001**
Yes	1.93 (1.31–2.75)	
No	2.62 (2.00–3.25)	
Was the type of online assessment successful?		0.001**
Yes	1.87 (1.31–2.56)	
No	3.00 (2.50–3.62)	

*Kruskal_Wallis (*H*)

**Mann_Whitney (*U*)

## Discussion

The COVID-19 pandemic has caused an unpredictable and unprecedented wave of closures of academic facilities, affecting millions of HEI students worldwide. Many academic institutions have been forced to take difficult decisions and implement modifications to their teaching and learning activities in order to continue delivering courses while avoiding the risk of virus transmission associated with students attending campuses.

The present study documents perceptions of student-centred active learning delivered through full distance learning during the pandemic period among a group of undergraduate students enrolled in various programmes at HCT campuses across the UAE. The HCT was already providing many online learning methods and tools for both teachers and students, but most of these were not fully utilized before the COVID-19 pandemic. During the pandemic, full distance learning was adopted, whereby online group discussions and new online assessments were carried out synchronously through the online communication platform known as BBL-Ultra. This study is the first to report on students’ perceptions of the effectiveness of the fully online learning methods and their learning satisfaction during the COVID-related restrictions.

### Student fatigue during e-learning

Participating students reported moderate fatigue levels when engaged in e-learning during the COVID-19 quarantine period. All subscales indicated low fatigue levels associated with e-learning. However, a significant percentage of students stated that they always felt fatigued more than five days per week. Possible reasons for these results, which are congruent with previous studies [10, 14, 15, 20], are the cumulative psychological and physiological burden experienced due to the unprecedented experiences associated with COVID-19 in the personal, social and academic domains. Results for the different items of the three scales indicate that students perceived the e-learning process to engender a burden that could negatively impact their psychological status and academic achievements.

Quality of course design to fit online learning and course instructor’s redness to provide quality feedback are factors found to increase student satisfaction and academic performance during the pandemic [21]. Our study revealed that students experienced lower fatigue levels if they were more satisfied with online assessment and had improved academic performance. Future studies should investigate factors escalating these burdens in more depth and seek solutions to minimize or prevent fatigue.

### Students’ psychological well-being during the COVID pandemic and quarantine

Participants in this study reported poor psychological well-being due to quarantine. This result was expected, as students were required to fully accept unpredictable changes and adapt to them immediately. Similar findings are reported by Cao and colleagues [1], who state that college students in China reported moderate to high anxiety levels and suffered many psychological effects of COVID-19 and the subsequent quarantine. One explanation for this might be the extended time spent working alone on computer screens, accompanied by the anxiety of being quarantined, adapting to an unprecedented situation and absorbing news about the numbers of confirmed COVID-19 cases or resultant deaths. Lack of necessary infrastructure, limited communication with the teacher, lack of face to face communication with colleagues, and low students’ motivation to engage with online learning were potential factors that affected students’ physical and mental well-being during the pandemic [22, 23].

Students taking part in this study were preoccupied with the fear of COVID infection, resulting in feelings of depression or anxiety which could be related to their reports of insomnia and lack of motivation. Similar results have recently been reported, where students in various programmes and at several academic levels reported moderate to severe levels of anxiety or mental health concerns [10, 24, 25]. Further, various factors were reported in the literature as stressors contributing to feelings of anxiety or depression during the pandemic, such as poor economic status [14]. Although exploring the factors/stressors contributing to anxiety goes beyond the purpose of this study, it is worth noting that students pursuing their studies online while being quarantined at home are likely to face many risks that would negatively impact their psychological status and mental well-being. Possible stressors are having a family member with a confirmed COVID-19 infection at home, changes imposed on their daily routines and fear of academic delays. Indeed, it is strongly recommended that educational institutions and authorities provide students with continuous support, ensuring the availability of the necessary emotional support mechanisms and counselling services, which should be available and accessible per each institution’s guidelines. Redesigning curricula with a more focused student-centred approach for online delivery, online academic support sessions, and implementing counselling and mental health programs are possible suggestions to care for at-risk students because of the pandemic.

This study has revealed a significant psychological effect of the COVID-19 pandemic on the teaching and learning of HCT students. While the crisis has been stressful for students, they are hoping, in common with other community members, that it will eventually end, that restrictions will be eased and that their lives will return, albeit gradually, to a pre-COVID normality. Therefore, in addition to sustaining students’ emotional and psychological well-being, offering stress counselling and applying the hybrid model of teaching which the HCT has announced, it is essential to continue to provide a reliable educational environment and to prepare a safe future for students. HCT students are familiar with e-learning but did not experience fully online delivery until the restrictive quarantine measures came into force. Any class of students will have diverse learning styles and it will thus be very challenging for the teacher to adapt and accommodate e-learning methods and tools to best meet their collective needs.

## Conclusion

The study reveals that new assessment approaches which emerged during home confinement reduced students’ perception of stress and of impaired lifestyle. However, students still had a considerable burden of psychological distress, requiring further preventive measures to maintain their psychological well-being during future outbreak events. Educational institutions should consider additional strategies to improve students’ preparedness for online teaching, which could help maintain their psychological well-being.

One of the main limitations of this study is its cross-sectional design; while this suited the present purpose, a future longitudinal study would be highly recommended to determine the long-term impacts on students’ health of the COVID-19 crisis. On the other hand, the large sample size among multiple colleges involved in the study is considered a strong point, upholding the generalizability of the findings and enabling the drawing of solid conclusions about the current situation in UAE universities on which to ground a possible national crisis strategy.

Fatigue levels were measured using a version of the Copenhagen Burnout Inventory, modified to fit the purpose of investigating online learning during COVID-19 quarantine. Although the three subscales as modified were found to have good internal consistency, further research could be conducted to confirm the internal validity of the scale. Finally, a further study could employ a more complex analytical approach to determine the confounding variables for students’ psychological well-being and the mediating effect of fatigue level.

## Data Availability

The datasets used and/or analysed during the current study are available from the corresponding author on reasonable request.
